# Mir-153-3p Modulates the Breast Cancer Cells’ Chemosensitivity to Doxorubicin by Targeting KIF20A

**DOI:** 10.3390/cancers15061724

**Published:** 2023-03-11

**Authors:** Khalil Ur Rahman, Shuo Yang, Nasir Azam, Zhen Yuan, Jiawen Yu, Chunhui Zhao, Bin Feng

**Affiliations:** 1 Department of Biotechnology, College of Basic Medical Sciences, Dalian Medical University, Dalian 116044, China; 2 Department of Hematology, The First Affiliated Hospital of Dalian Medical University, Dalian 116011, China; 3 College of Life Sciences, Liaoning Normal University, Dalian 116029, China

**Keywords:** miR-153-3p, chemosensitivity, breast cancer, doxorubicin, KIF20A

## Abstract

**Simple Summary:**

Breast cancer is a disease that begins in the cells of the breast. Although it is most common in women, it can also occur in men, though this is rare. Early detection and treatment are crucial to improving outcomes and survival rates. Risk factors include age, family history, certain gene mutations, prior history of breast cancer, exposure to estrogen, and lifestyle habits like alcohol consumption and inactivity. Symptoms include changes in the skin on the breast, such as dimpling, lumps or thickening, changes in size or shape, and discharge from the nipple. Diagnosis is often made through a clinical breast exam, mammogram, biopsy, and possibly additional imaging tests. Treatment options include surgery, chemotherapy, radiation therapy, hormone therapy, and targeted therapy. The choice of treatment method depends on the stage and characteristics of cancer.

**Abstract:**

Breast cancer is considered the solid tumor most sensitive to chemotherapy. However, it can become resistant to various chemotherapeutic drugs, including doxorubicin, which triggers cell death by intercalation between DNA bases, free radical formation, and topoisomerase II inhibition. When drug resistance develops, several miRNAs are dysregulated, suggesting that miRNAs may play a significant role in resistance formation. In the current study, we investigated how doxorubicin sensitivity of breast cancer cells is affected by miR-153-3p and its target gene. The MTT method was used to determine the chemo-sensitizing effect of miR-153-3p on doxorubicin in MCF-7 and MDA-MB-231 cell lines. Results of Western blot and dual luciferase confirmed that miR-153-3p targets KIF20A and decreases its expression. Transwell and flow cytometry experiments showed that miR-153-3p and doxorubicin together had higher effects on MCF-7 and MDA-MB-231 cell proliferation, migration, and invasion, as well as increasing apoptosis and arresting cells in the G1 phase. Proteins related to apoptosis and the cell cycle exhibited the same tendency. Intracellular vesicle formation was inhibited and RAB26 was also downregulated by treatment with miR-153-3p alone or in combination with doxorubicin. Doxorubicin’s ability to suppress tumors may be enhanced by miR-153-3p, according to in vivo studies. According to our findings, miR-153-3p has a direct effect on KIF20A and may regulate the formation of intracellular vesicles, which in turn makes breast cancer cells more susceptible to doxorubicin.

## 1. Introduction

Breast cancer is a complex disease that arises from the uncontrolled growth of breast cells and is a major cause of death in females. The exact mechanisms of breast cancer development and progression are not fully understood, but several factors have been implicated. One of the main mechanisms involves mutations in genes that regulate cell growth and division. These mutations can lead to the formation of abnormal cells that divide uncontrollably and accumulate to form a tumor. The most commonly mutated genes in breast cancer include *TP53*, *BRCA1*, and *BRCA2* [1,2]. Hormonal factors also play a role in the development of breast cancer. Exposure to high levels of estrogen, a hormone that stimulates cell growth, can increase the risk of breast cancer. Therefore, breast cancer is more common in women who have a long history of exposure to estrogen, such as those who start menstruating at an early age, have late menopause, or use hormone replacement therapy. The stimulation of signaling pathways that control cell growth and survival is another way that breast cancer develops. Abnormal activation of these pathways, such as the HER2/neu pathway, can lead to uncontrolled cell growth and the development of breast cancer [3]. In addition, lifestyle factors, such as diet, exercise, and exposure to environmental pollutants, have also been reported for breast cancer. A combination of genetic, hormonal, and environmental factors is thought to contribute to the development of this disease. Although several treatments are available, including surgical resection, chemotherapy, and radiotherapy, at an advanced level of cancer its prognosis remains poor [4].

Breast cancer is considered the type of solid tumor most sensitive to chemotherapy, but it can relapse and become resistant to various chemotherapeutic drugs. Doxorubicin, an “anthracycline” antibiotic prescribed for solid and blood tumors, is the most effective single-agent treatment. The mechanisms by which doxorubicin triggers cell death include intercalation between DNA bases during the replication process, free radical formation [5], and topoisomerase II inhibition [6,7]. An inhibitor of topoisomerase II mainly prevents broken DNA fragments from rejoining, and the efficiency of such inhibitors depends on the level of topoisomerase II expression within the cells. An identified mechanism of resistance to a topoisomerase II inhibitor is the altered expression and enhanced efflux level of topoisomerase II. Previously, patients exposed to doxorubicin had a 50% probability of unsuccessful outcomes of recurrence of breast cancer due to metastasis, with the mechanisms involved in disease progression including degradation of the extracellular matrix, aberrant adhesion of cancerous cells, epithelial-to-mesenchymal transition (EMT) invasion and angiogenesis [8].

Various cellular processes, such as embryo development, angiogenesis and cell cycling, migration, and apoptosis, are regulated by microRNAs. Dysregulated miRNA levels lead to oncogenesis either by suppressing tumor suppressor genes or by activating oncogenes [9]. miR-153 is a microRNA that has been shown to play a role in the development and progression of cancer. By binding to messenger RNA and preventing its translation into protein, this small non-coding RNA molecule regulates gene expression. In cancer cells, miR-153 has been found to target and suppress multiple genes involved in cell growth, survival, and invasion, making it a potential therapeutic target. Recently, the role of miR-153 in different cancers has become an interest of different research groups. In bladder cancer, miR-153 decreased tryptophan catabolism and inhibited angiogenesis by targeting *IDO1* [10]. In lung cancer, overexpression of miR-153 influenced *ABCE1* expression to sensitize resistance against gefitinib [11]. Meanwhile, miR-153 was reported to suppress the proliferation and migration of lung cancer cells by affecting the AKT pathway [12]. In glioblastoma, the chromatin-modifying drugs upregulated miR-153, which indicates its association with chromatin and oncogene suppression [13]. In breast cancer cells, miR-153 downregulated *HECTD3*, which promotes apoptosis [14] and inhibits metatherian, which in turn suppresses the EMT. By targeting *RUNX*2, miR-153 overexpression also reduced EMT, proliferation, migration, and invasion [15]. According to reports, miR-153 is frequently downregulated in various cancers, including breast, lung, and prostate cancer, and its restoration has been linked to a reduction in tumor development and better patient outcomes. Further research is needed to fully understand the role of miR-153 in cancer and to develop effective strategies for targeting this microRNA in the clinic.

Considering these previous studies, miR-153 may act as an “onco-suppressor” in cancers, so it is crucial to understand its regulatory mechanisms to develop an effective treatment. It is unclear how miR-153 contributes to doxorubicin resistance during treatment for breast cancer. This work aimed to examine the molecular mechanism by which miR-153-3p and its target gene overcome chemotherapeutic drug resistance in breast cancer cells as well as the impact of miR-153 on doxorubicin chemosensitivity in breast cancer cells.

## 2. Materials and Methods

### 2.1. Transfection of Cells

The CAS-Cell Bank in Shanghai, China, provided the MCF-7 and MDA-MB-231 human breast cancer cell lines. The cells were cultured and maintained in Dulbecco’s modified Eagle medium (Hyclone, Logan, UT, USA) with 10% fetal bovine serum, 100 U/L penicillin antibiotics, and 100 mg/L streptomycin antibiotics. The cultured cells were incubated at 37 °C in the presence of 5% CO_2_. GenePharma Co., Ltd (Shanghai, China). created the miR-153-3p mimics (5′-UUGCAUAGUCACAAAAGUGAUC-3′), inhibitor mimics (5′-GAUCACUUUUUUUUUUUUUUGCAA-3′), and negative control mimics (NC) (Shanghai, China). According to the manufacturers’ instructions, the cells were transfected using lipofectamine 2000 (Life Technologies Corp., Carlsbad, CA, USA, Cat# 11668-019) and mimic complexes.

### 2.2. Response of Doxorubicin Chemosensitivity against miR-153-3p

To permit overnight attachment to a 96-well plate, approximately 3000 cells were seeded in each well. To determine the IC_50_ values of miR-153-3p mimics and doxorubicin (Dox, Aladdin Reagent Co. Ltd., Shanghai, China, Cat# D396221), the cells were transfected with different concentrations of miR-153-3p mimics, inhibitor and NC ranging from 4 to 20 nM, and Dox concentrations ranging from 0.2 to 3.2 μM. The MTT assay, as previously described, was used to determine cell viability after a 24-h incubation period [16]. The dose-response curve was used to calculate the IC_50_ value of Dox and miR-153-3p.

### 2.3. Immunofluorescence of Ki67 and CD63

The breast cancer cells were treated for 24 h with miR-153-3p mimics, inhibitors, NC, Dox, and miR-153-3p+Dox before being fixed for ten minutes in 4% paraformaldehyde and 0.2 percent Triton X-100. The cells were washed three times with PBS, incubated with antibodies against Ki67 and CD63 (Abcam, Cambridge, UK) overnight at 4 °C, and then blocked with 1% BSA for an hour. The cells were then exposed to a goat anti-rabbit secondary antibody from Life Technologies, Carlsbad, CA, USA, that was 594-conjugated with Alexa fluorescence for an hour at 37 °C. After being cleaned three times with PBS, the nuclei were stained for five minutes with DAPI (Life Technologies). The fluorescent signals were then examined using an Olympus IX71 fluorescence microscope.

### 2.4. Dual Luciferase Reporter Assay

The seed sequence of miR-153-3p in the 3′-UTR of the KIF20A (Kinesin family member 20A is a protein that plays a role in various cellular processes such as cell division, cytokinesis, and intracellular transport) gene was predicted by Target Scan and miRanda. Total RNA was extracted using the RNAiso Plus reagent (Takara Bio Inc., Shiga, Japan, Cat# 9108), cDNA was created using a total of 1 g of total RNA, plasmids for the dual luciferase reporter experiment were created, and the wild-type 3′-UTR of the KIF20A gene was amplified by using P1 (5′-CCGGAGCTCAAAGAGAAGAGCAGTCATGGC-3′) and P2 (5′-CCGGTCGACTGATTTTGCTACATTTGGAATTC-3′) primers (the restriction sites of Sac*I* and Sal*I* are underlined in the primer sequences). Overlapping PCR was used to mutate the binding site of miR-153-3p in the 3′-UTR of the KIF20A gene. P3 (5′-ACTGTTTTTGTGTGCCGAGAAATCATATAAGTAAAT-3′) and P2, P4 (5′-ATTTACTTATATGATTTCTCGGCACACAAAAACAGT-3′) and P1 were used to amplify the mutated fragments of the 3′-UTR. Then, the two mutated fragments were mixed, and PCR was performed with P1 and P2 to obtain the mutated 3′-UTR. The wild-type and mutant 3′-UTR fragments were purified and digested with the restriction enzymes separately. The fragments were then inserted into pGLO vectors predigested with the same enzymes to result in plasmids pGLO-KIF-WT (wild-type) and pGLO-KIF-MUT (mutated type). Sequencing of the recombinant plasmids was done through Sangon Biotech, Shanghai, China.

To allow attachment, approximately 4000 293T cells were incubated overnight by using a 96-well plate. The cells were then co-transfected with miR-153-3p mimics or NC, depending on the situation, and transfected with pGLO-KIF-WT and pGLO-KIF-MUT. The Dual-Luciferase Assay Kit (Promega, Madison, WI, USA, Cat#E1910) was used in accordance with its normal protocol to carry out the luciferase assay. After transfection and incubation for 24 h, this system was used to quantify the amounts of Renilla and firefly luciferases consecutively. Three independent experiments were carried out to obtain the mean value of the Renilla/firefly luciferase ratio.

### 2.5. Cell Migration and Invasion Assays

The MCF-7 and MDA-MB-231 cells were planted in a six-well plate with roughly 2 × 10^5^ cells per well for overnight incubation. Next, miR-153-3p mimics, inhibitors, or NC were transfected into the cells at a dose of 20 nM for each. The combination treatment with 20 nM miR-153-3p and 1.6 μM Dox was used to check the effects on migration and invasion. The cells were trypsinized and collected after 24 h of treatment. Around 2 × 10^4^ cancer cells were seeded in 200 μL of DMEM containing 1% FBS in the upper chamber of the transwell cups of a 24-well plate made of polycarbonate with 8 μm pores (Corning Inc., Corning, NY, USA). In the lower chamber, a chemoattractant consisted of a 500 μL medium containing 10% FBS. The chambers were washed three times with PBS after a 24 h incubation, and the inner surfaces of the cups were wiped with cotton swabs gently. Methanol was used to fix the cells for 20 min and Giemsa solution was used for staining. The cells were counted under an inverted microscope with a 40× objective lens. The chambers were coated with Matrigel (BD Biosciences, San Jose, CA, USA) at a 1:4 dilution in free DMEM for the invasion assay, and they were then incubated for 1 h at 37 °C. The subsequent steps were identical to those used for the migration assay.

### 2.6. Flow Cytometry of Apoptosis and Cell Cycle Arrest

The MCF-7 and MDA-MB-231 cells were cultivated in 6 cm plates and treated with doxorubicin, miR-153-3p, and the combination. Doxorubicin was present in concentrations of 1.6 µM and 20 nM, respectively, of inhibitor, NC, and miR-153-3p. The cells were trypsinized and collected after 24 h of treatment. Annexin V-apc detection kit (KeyGEN BioTECH, Shanghai, China, Cat# KGA107) was used to analyze the apoptotic and cell cycle arrested percentages of MCF-7 cells treated with miR-153-3p mimics, NC, inhibitor, Dox, and miR-153-3p mimics+Dox. EDTA-free trypsin was used to collect the treated cells for the apoptosis assay. After being washed twice with cold PBS, the cells were resuspended in a binding buffer and stained for up to 15 min with annexin V-FITC and propidium iodide (PI). The percentage of annexin V-FITC-positive cells was determined using a CytoFLEX flow cytometer from Beckman Coulter, Inc., CA, USA. For the cell cycle analysis, the treated cells were harvested using trypsin. The cells were resuspended in 70% ethanol after being washed twice with PBS, and they were then kept at 4 °C overnight for storage. RNase A was used to treat the cells for 30 min at room temperature, and they were then treated with PI for 30 min, in the dark, at 4 °C. The processed samples were transferred to ice and analyzed using the flow cytometer.

### 2.7. Detection of Proteins through Western Blot

After being subjected to treatment with miR-153-3p mimics, NC, inhibitor, Dox, or miR-153-3p mimics+Dox for a period of 24 h, the MCF-7/MDA-MB-231 cells were lysed with RIPA buffer (Sigma, St. Louis, MO, USA) for the purpose of protein extraction. A PVDF membrane was loaded with proteins in equal amounts for SDS-PAGE. The primary antibodies from SanYing Biotechnology (Wuhan, China) against KIF20A, PCNA, CDK4, BAX, Bcl-2, Cytochrome C, Rab26, and PARP1 (all rabbit polyclonal) were incubated with all blots for an entire night at 4 °C. Before applying the horseradish peroxidase-conjugated secondary antibodies to the blots, the membranes were washed three times with TBST after purchasing β-actin (mouse monoclonal). An ECL kit from Thermo Scientific (Cat# 32106) and a ChemiDoc MP imaging system from Bio-Rad, Hercules, CA, USA, were used to see the signals on the membranes. Image J was used to measure the signal strength, and the ratio of the target protein to the internal control actin was used to represent the difference in relative expression.

### 2.8. Tumorigenicity in Nude Mice

Female athymic nude mice (BALB/c, 12–15 g), 4–6 weeks old, were purchased from Beijing Weitong Lihua Experimental Animal Technology Co., Ltd. The mice were bred and housed in the SPF laboratory animal room system in the Medical Experimental Animal Center of Dalian Medical University. All the following experimental operations were carried out in accordance with the regulations approved by the Animal Experiment Ethics Committee of Dalian Medical University. In order to construct a xenograft model of breast cancer, MCF-7 cells in the logarithmic growth phase were taken, and the concentration of viable cells was adjusted to 5 × 10^7^ cells/mL. The cells were subcutaneously inoculated on both sides of the abdomen of nude mice (0.1 mL/only), with a total of 20 only.

After tumor cell inoculation, the tumor growth was observed every day. After 10 days, when tumors became visible, the twenty transplanted nude mice were randomly divided into four groups (*n* = 5 each), and then miR-153-3p agomir (Shanghai GenePharma Co., Ltd., Shanghai, China) and Dox were administered as follows: Group 1, miR-NC agomir was injected directly into transplanted tumors once every second day, and each tumor was injected with a dose of 0.5 nmol/20 μL PBS each time; Group 2, miR-153-3p agomir 0.5 nmol/20 μL PBS was injected into each tumor every second day; Group 3, Dox 1.5 mg/kg i.p. was injected into each tumor every second day; and Group 4, Dox 1.5 mg/kg i.p.+miR-153-3p agomir 0.5 nmol/20 μL PBS was injected into each tumor every second day. Changes in the tumor volume were recorded during the treatment period, and the experimental period was 35 days from the inoculation of MCF-7 cells to the end. Tumor volume was calculated as V = (length × width^2^)/2. Mice were sacrificed and the tumors were excised and photographed.

### 2.9. Statistical Analysis

For quantitative data analysis, GraphPad Prism was used. Student’s *t*-test (* *p* < 0.05; ** *p* < 0.01; *** *p* < 0.001) was applied in the whole study.

## 3. Results

### 3.1. Doxorubicin-Sensitive Breast Cancer Cells Are Caused by the Overexpression of miR-153-3p

**Figure 1:**
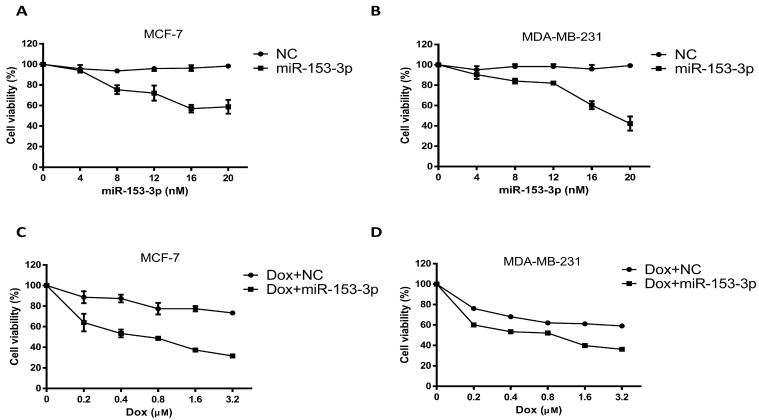
Overexpression of miR-153-3p enhances the chemosensitivity of breast cancer cells to doxorubicin. Cell viability was analyzed after 24 h via the MTT assay in MCF-7 (**A**,**C**) and MDA-MB-231 (**B**,**D**) cells.

The breast cancer cells were transfected with different concentrations of miR-153-3p mimics for 24 h. The cell viability decreased significantly as the concentrations of miR-153-3p mimics increased (Figure 1A,B). At 20 nM concentration, the cell viabilities of MCF-7 and MDA-MB-231 cells were about 58% and 42%, respectively. When breast cancer cells were treated with doxorubicin combined with 20 nM miR-153-3p mimics, a significant further decrease in cell viability was observed as the concentration of doxorubicin increased. The viability percentage dropped from 72.6% to 31% in MCF-7 cells and from 59% to 36% in MDA-MB-231 cells with 3.2 µM of doxorubicin (Figure 1C,D). The combined treatment showed that miR-153-3p efficiently sensitized both MCF-7 and MDA-MB-231 cells to doxorubicin. In the next experiments, miR-153-3p mimics at 20 nM and doxorubicin at 1.6 µM were used for independent or combination treatment.

### 3.2. Effects of miR-153-3p and Doxorubicin on Ki67 Expression

**Figure 2:**
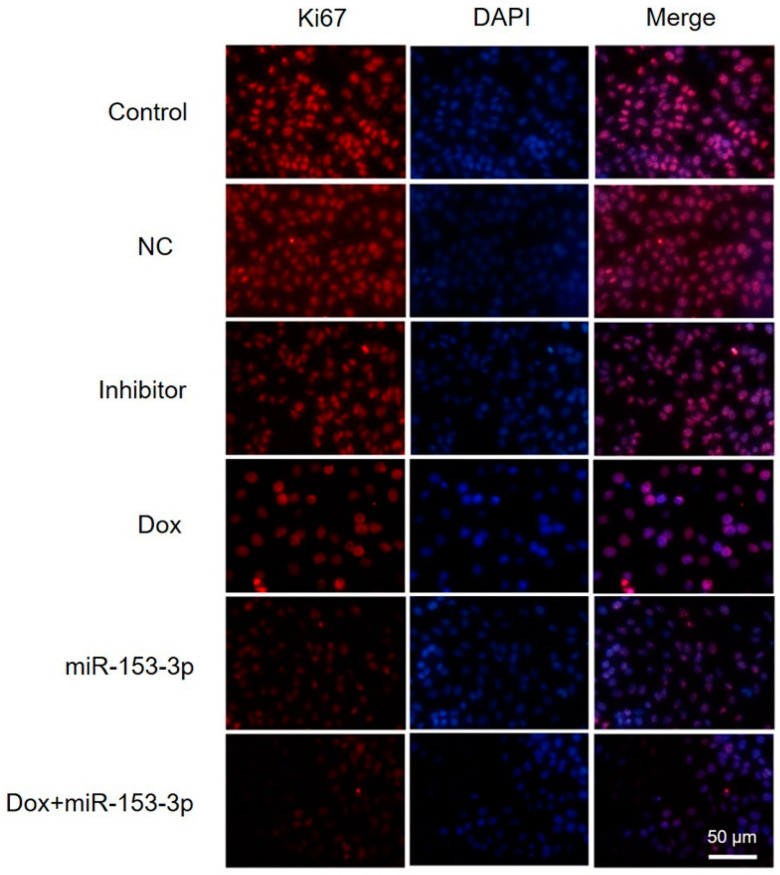
Immunofluorescence indicating Ki67 expression in MCF-7 cells after miR-153-3p and Dox treatment. MCF-7 cells were transfected with NC, inhibitor, miR-153-3p or Dox, and miR-153-3p+Dox for 24 h. Scale bar = 50 μm.

After MCF-7 cells were given miR-153-3p, doxorubicin, or both, a fluorescence microscope was used to look for Ki67 protein expression. Both miR-153-3p and doxorubicin effectively attenuated Ki67 expression compared with the control and NC or inhibitor (Figure 2). The fluorescence in the combination treatment group with miR-153-3p mimics and doxorubicin showed a significantly decreased signal, and the expression level was lower than that with treatment by miR-153-3p or doxorubicin alone. In this regard, miR-153-3p mimics enhanced the inhibitory effect of doxorubicin on breast cell proliferation.

### 3.3. KIF20A Is a Direct Target Gene of miR-153-3p and Is Inhibited by miR-153-3p and Doxorubicin

**Figure 3:**
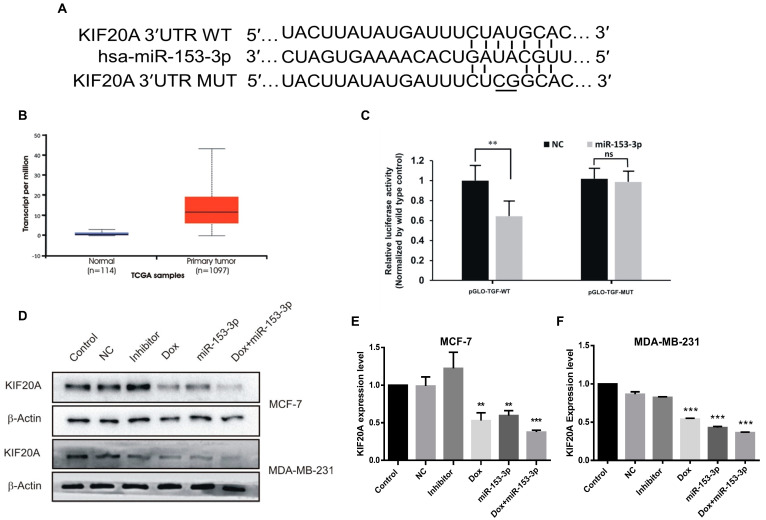
KIF20A is a direct target gene of miR-153-3p and was downregulated by miR-153-3p. (**A**) Binding sites of miR-153-3p in the 3’-UTR of KIF20A. (**B**) KIF20A expression analysis in the TCGA database. (**C**) Quantification of relative luciferase activity in wild-type and mutant vectors. Data is the mean ± SD of three separate experiments. (**D**) Expression levels of KIF20A were detected by Western blotting (original blot see Supplementary File S1). (**E**,**F**) Quantitative analysis of KIF20A expression levels in MCF-7 cells and MDA-MB-231 respectively (ns: not significant; ** *p* < 0.01; *** *p* < 0.001.

The target gene predicting algorithms, miRanda and Target Scan, indicated that the KIF20A gene is a potential target of miR-153-3p, and the binding site to the seed sequence of miR-153-3p could be found in the 3′-UTR of KIF20A (Figure 3A). According to the TCGA database analysis, the expression of KIF20A was relatively higher in the tumor than in normal tissues in breast cancer (Figure 3B). To confirm the relationship between the target gene (*KIF20A*) and miR-153-3p, the seed sequence (CUAUGCA) was mutated to CUCGGCA, and the dual luciferase reporter assay was performed. Co-transfection of miR-153-3p and pGLO-KIF-WT caused a significant decrease in fluorescence intensity compared to the control groups, indicating that KIF20A is a direct target of miR-153-3p (Figure 3C). Western blotting results showed that the combination of miR-153-3p mimics and doxorubicin enhanced the suppression of KIF20A expression significantly as compared to doxorubicin or miR-153-3p mimics alone in MCF-7 and MDA-MB-231 cells. The control, NC, and inhibitor groups had insignificant effects on the expression of KIF20A (Figure 3D–F).

### 3.4. Overexpression of miR-153-3p Enhances Doxorubicin-Induced Apoptosis

**Figure 4:**
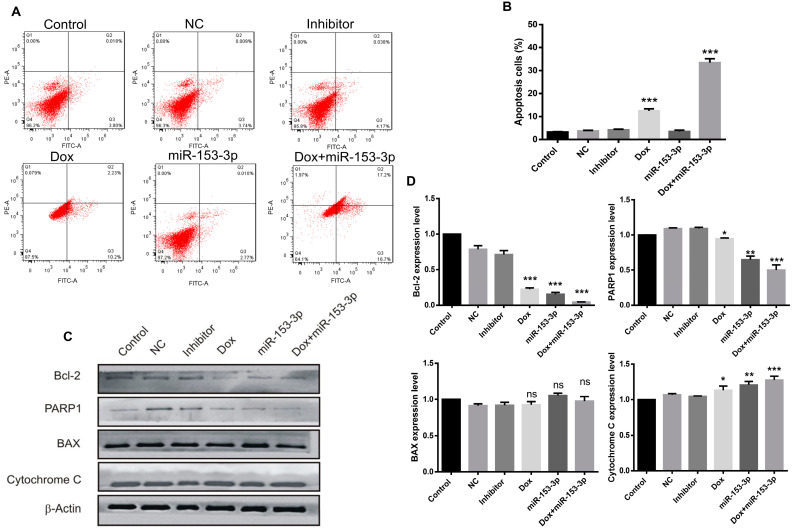
Overexpression of miR-153-3p or Dox treatment induces apoptosis in MCF-7 cells. (**A**) Cell apoptosis was evaluated by flow cytometry after 24 h of treatment. (**B**) Comparison of the percentages of apoptotic cells after different treatments. (**C**) Expression levels of Bcl-2, PARP1, BAX, and cytochrome C were detected by Western blot (original blot see Supplementary File S1). (**D**) Quantification of apoptosis-related proteins (ns: not significant; * *p* < 0.05; ** *p* < 0.01; *** *p* < 0.001).

**Figure 5:**
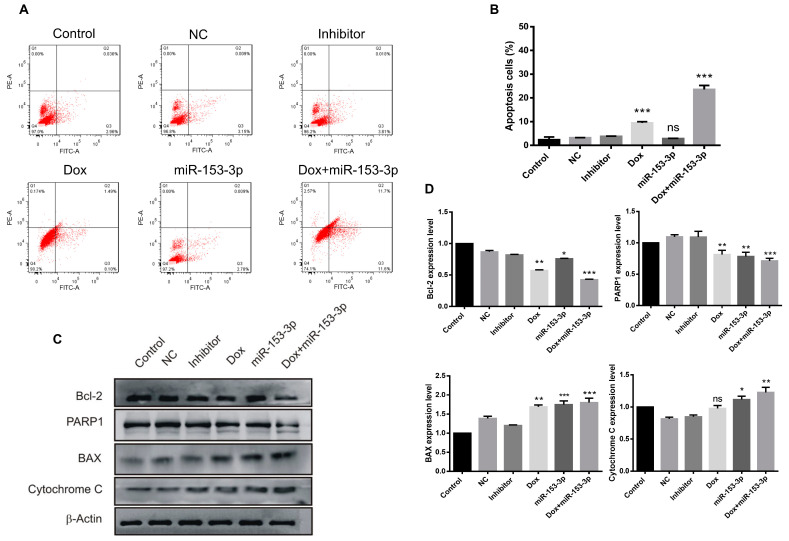
Overexpression of miR-153-3p or Dox treatment induces apoptosis in MDA-MB-231 cells. (**A**) Cell apoptosis was evaluated by flow cytometry after 24h of treatment. (**B**) Comparison of the percentages of apoptotic cells after different treatments. (**C**) Expression levels of Bcl-2, PARP1, BAX, and cytochrome C were detected by Western blot (original blot see Supplementary File S1). (**D**) Quantification of apoptosis-related proteins (ns: not significant; * *p* < 0.05; ** *p* < 0.01; *** *p* < 0.001).

To investigate the effects of miR-153-3p, doxorubicin, and the combination on apoptosis, flow cytometry analysis was performed on both cancer cell lines. In MCF-7 cells the percentage of early and late apoptotic cells was 12.43% in doxorubicin-treated cells, while when combined with miR-153-3p treatment, the number of apoptotic cells reached 33.9% (Figure 4A,B). Similar enhanced results were observed in MDA-MB-2321 cells with combination treatment, compared with doxorubicin alone, the number of apoptotic cells increased from 9.59% to 23.3% (Figure 5A,B).

The expression of apoptosis-related proteins was also checked in breast cancer cells. Compared with miR-153-3p or doxorubicin treatment alone, the levels of Bcl-2 and PARP1 were downregulated, and Cytochrome C was upregulated significantly in cells treated with the combination of miR-153-3p and doxorubicin as shown in Figure 4C,D, for MCF-7 cells and Figure 5C,D for MDA-MB-231 cells. No obvious additive effect on the Bax protein expression level was observed in the MCF-7 cell line (Figure 4C,D).

### 3.5. Effects of miR-153-3p on Cell Migration and Invasion

**Figure 6:**
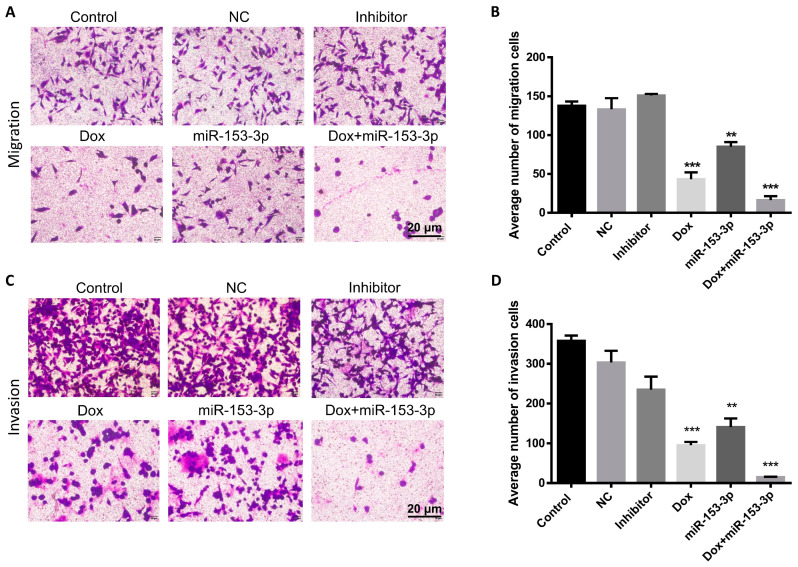
miR-153-3p, Dox, and miR-153-3p+Dox treatment inhibited migration (**A**,**B**) and invasion (**C**,**D**) of MCF-7 cells (** *p* < 0.01; *** *p* < 0.001).

**Figure 7:**
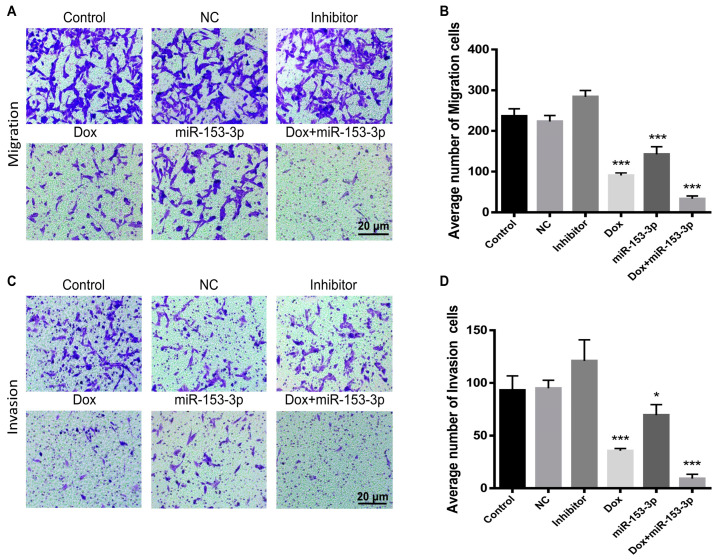
miR-153-3p, Dox, and miR-153-3p+Dox treatment inhibited migration (**A**,**B**) and invasion (**C**,**D**) of MDA-MB-231 cells (* *p* < 0.05; *** *p* < 0.001).

Transwell assays were used to see how miR-153-3p and doxorubicin combined treatment affected breast cell migration and invasion. Overexpression of doxorubicin and miR-153-3p effectively inhibited invasion and migration in comparison to the controls. In addition, in MCF-7 and MDA-MB-231 cells, the synergistic effect of doxorubicin and miR-153-3p was significantly more significant than the effect of each treatment on its own. The migration, invasion and quantitative analysis of MCF-7 cells and MDA-MB-231 cells are shown in Figure 6A–D and Figure 7A–D, respectively.

### 3.6. Effect of miR-153-3p on Cell Cycle

**Figure 8:**
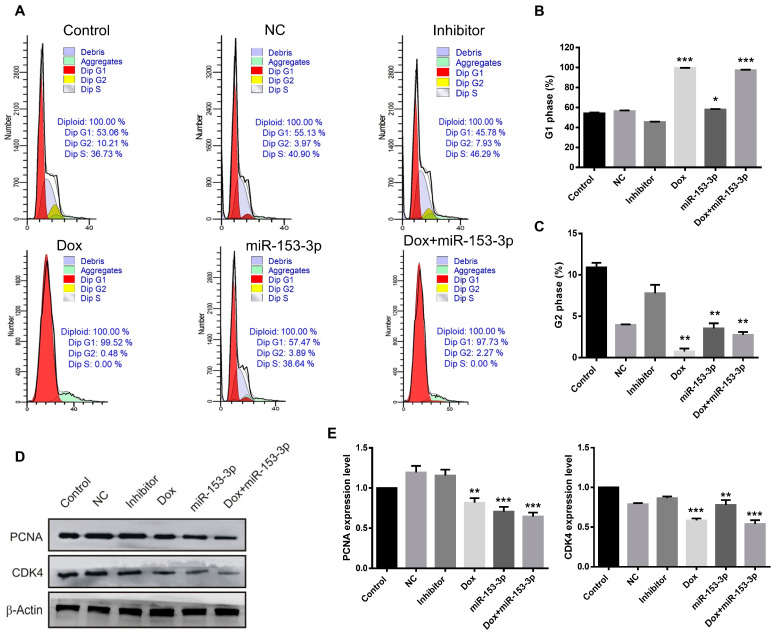
Effects of miR-153-3p, Dox, and miR-153-3p+Dox treatment on the cell cycle in MCF-7 cells. (**A**) Cell cycle phase distribution by flow cytometry. (**B**,**C**) Quantification of the G1 and G2 phases. (**D**,**E**) Western blotting of CDK4 and Cyclin D1 and their quantification (* *p* < 0.05; ** *p* < 0.01; *** *p* < 0.001, original blot see Supplementary File S1).

**Figure 9:**
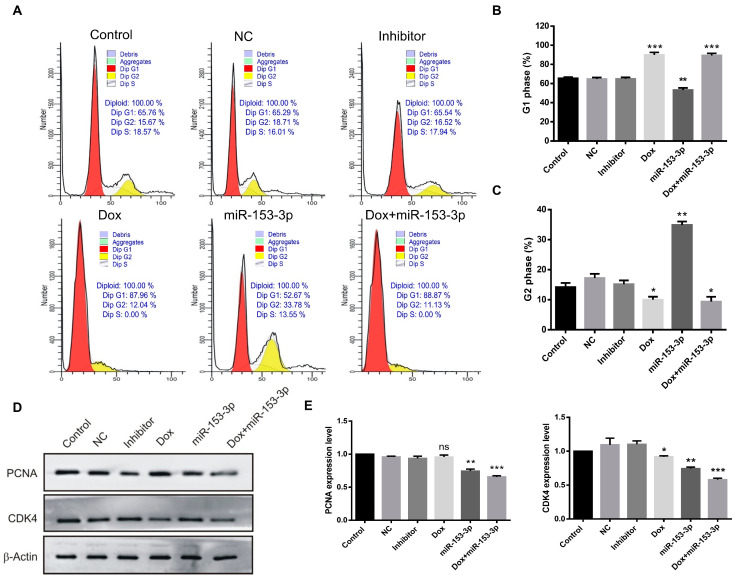
Effects of miR-153-3p, Dox, and miR-153-3p+Dox treatment on the cell cycle in MDA-MB-231 cells. (**A**) Cell cycle phase distribution by flow cytometry. (**B**,**C**) Quantification of the G1 and G2 phases. (**D**,**E**) Proteins analysis of CDK4 and Cyclin D1 (ns: not significant; * *p* < 0.05; ** *p* < 0.01; *** *p* < 0.001, original blot see Supplementary File S1).

In order to determine the arrested phase, the treated groups were analyzed by flow cytometry. In the MCF-7 cells, the percentage of cells in the G1 phase was 99.52% after doxorubicin treatment and 97.73% after combination with miR-153-3p treatment. The percentages of cells treated with control, NC, and inhibitor in the G1 phase were 53.06%, 55.13%, and 45.78%, respectively (Figure 8A–C). In the MDA-MB-231 cells, the percentage of cells in G1 phase arrest was 87.96% after doxorubicin treatment, and 88.87% after combination treatment with miR-153-3p, while the percentages of cells in the G1 phase after control, NC and inhibitor treatment were 65.76%, 65.29%, and 65.54%, respectively (Figure 9A–C). Although the miR-153-3p treated groups did not show an obvious increase in the G1 phase in the two cell lines, doxorubicin had a very strong effect on G1 phase arrest and miR-153-3p played a contributing role in cell cycle arrest in the G1 phase.

After miR-153-3p, doxorubicin, and combination treatment, variations in the expression of cell cycle-related proteins were further demonstrated by Western blotting. After treatment with miR-153-3p and doxorubicin on their own, CDK4 and PCNA levels decreased, while the combination had a significant down-regulatory effect, as depicted in Figure 8D,E and Figure 9D,E.

### 3.7. Effects of miR-153-3p Mimics on Intracellular Vesicle Packaging

**Figure 10:**
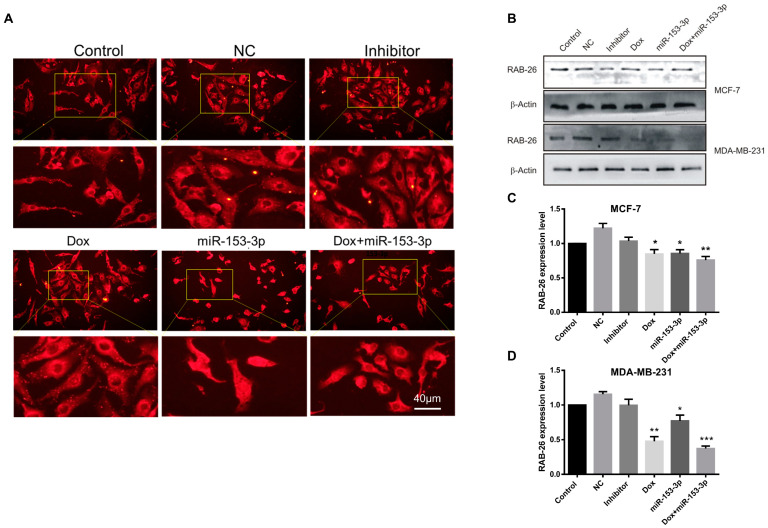
Immunofluorescence assay of CD63 and Western blotting of RAB26 after miR-153-3p and Dox treatment. (**A**) MCF-7 cells were transfected with NC, inhibitor, miR-153-3p or Dox, and miR-153-3p+Dox for 24 h. (**B**–**D**) Western blotting of RAB26 and its quantitative analysis in MCF-7 cells. Scale bar = 40 μm (* *p* < 0.05; ** *p* < 0.01; *** *p* < 0.001, original blot see Supplementary File S1).

To check the effect of miR-153-3p on intracellular vesicle formation, the cell-surface protein CD63 was used as an immunofluorescence marker. As shown in Figure 10, prominent vesicles could be clearly observed in the control, NC, and inhibitor-treated cells. Interestingly, doxorubicin did not affect the production of intracellular vesicles, while there was no trace of intracellular vesicles in miR-153-3p or combination-treated cells. In both MCF-7 and MDA-MB-231 cells, Western blot results showed that miR-153-3p, Dox, and Dox+miR-153-3p treated cells had significantly lower levels of RAB-26 expression—an important regulator of vesicular fusion and trafficking.

### 3.8. miR-153-3p Increases the Chemosensitivity of Dox In Vivo

**Figure 11:**
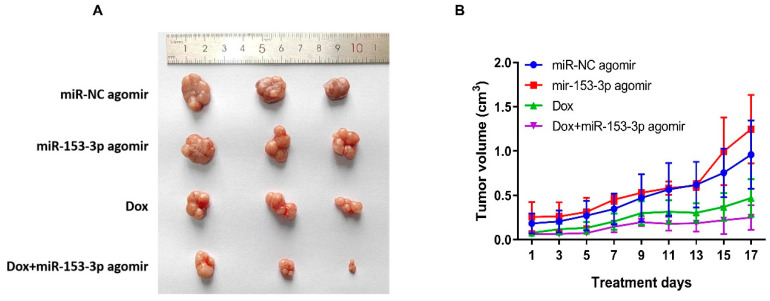
The tumor’s images after treatment with miR-NC agomir, miR-153-3p agomir, Dox, and Dox+miR-153-3p agomir (**A**). The size and volume curves of tumors treated with miR-NC agomir, miR-153-3p agomir, Dox, Dox+miR-153-3p agomir (**B**).

A subcutaneous xenograft model was established to analyze the role of miR-153-3p in regulating the Dox sensitivity of breast cancer cells in vivo. As shown in Figure 11A, compared with the Dox and miR-153-3p agomir treatment group alone, the Dox+miR-153-3p agomir group has a significant enhancement effect on inhibiting tumor growth. The tumor growth curve in Figure 11B is consistent with the effect. This result indicates that miR-153-3p can enhance the sensitivity of Dox in vivo.

## 4. Discussion

The spread of abnormal cells is the hallmark of a group of diseases known as cancer. Depending on the type of cells involved, it can manifest in a variety of ways and can occur in any part of the body including the lungs, breast, prostate, and colon [17]. Causes of cancer can include genetic mutations, exposure to certain substances or radiation, and certain lifestyle choices [18]. Early detection and proper treatment can improve outcomes for many people with cancer [19]. Surgery combined with adjuvant treatment including chemotherapy is currently the most effective strategy for breast cancer therapy. However, 30–70% of patients will develop recurrent or metastatic disease [20]. Despite the excellent role of doxorubicin in anti-tumor activity, intrinsic or acquired drug resistance limits the efficacy of doxorubicin-based treatment [21]. Even in patients who achieve the maximum amount of tumor cytoreduction [22,23], chemoresistance continues to be a key barrier to enhancing cure effectiveness, and the majority of therapies ultimately fail due to secondary recurrence, metastasis, and drug resistance [24]. However, the major problems with doxorubicin treatments are cardiotoxicity and the induction of multidrug resistance. The exact mechanisms of cardiotoxicity are not clear, but oxidative stress is one of the cardinal causes of doxorubicin-induced cardiotoxicity [25]. Multidrug resistance protein expression and other causes are the most frequently proposed underlying mechanisms contributing to doxorubicin resistance. Kumar et al. revealed that the interplay between the miR-495-3p, *TGF-β2*, and *FOXC1* may contribute to the regulation of multidrug resistance in metastatic breast cancer [26].

When drug resistance develops, multiple miRNAs were found to be dysregulated, which suggests that miRNAs may play a significant role in the resistance [27,28]. The miR-140 was reported to act as a tumor suppressor by targeting the *FEN1* gene which leads to repressing the DNA damage repair in breast cancer cells [29]. Long et al. found that myeloid cell leukemia-1 protein (MCL-1) was significantly overexpressed in MCF-7/DOXR cells and that miR-193b sensitized breast cancer cells to doxorubicin by targeting *MCL-1* [30]. Downregulation of miR-134 may also increase breast cancer doxorubicin resistance by controlling the ATP binding cassette C1 (*ABCC1*) gene [20]. Overexpression of the important efflux transporter multidrug resistance-associated protein 1 (MRP) typically results in chemoresistance in breast cancer. In MCF-7 cells, it was discovered that miR-145 had a negative effect on MRP1 regulation, and miR-145 made MCF-7 cells more sensitive to Adriamycin (ADR) by inhibiting MRP1 expression and causing intracellular doxorubicin accumulation [31]. Another study demonstrated that STAT5a may be a promising target to overcome resistance in the breast cancer [32].

Although miR-153 has multiple targets in different cancers, its relationship with doxorubicin resistance and KIF20A has not previously been clarified in breast cancer cells. In this study, we found miR-153-3p could target KIF20A to sensitize breast cancer cells to doxorubicin. KIF20A is a motor protein with microtubule plus end-directed motility. KIF20A may provide an anchorage site for Rab6-myosin IIA near the microtubule nucleating site, which involves the transport carriers from Golgi/trans-Golgi network membranes [33]. Microtubule motors play central roles in intercellular trafficking, force generation, and migration. Inhibition of KIF20A helps to soften the intracellular environment in both high- and low-grade bladder cancer. The cortical stiffness is highly correlated with the interaction between KIF20A and myosin IIA [34]. It is reported that paclitaxel targets KIF20A to drive abnormal spindle formation in mouse embryonic fibroblasts, and that deregulation of KIF20A leads to paclitaxel resistance [35]. KIF20A is also involved in taxane resistance in breast cancer cells [36]. The expression of KIF20A is relatively high in bladder cancer tissues, and patients with a high expression of KIF20A have a poor prognosis and a high rate of metastasis. Knockdown of KIF20A inhibits cell migration and proliferation in bladder cancer and prostate cancer [37]. In colorectal cancer, KIF20A provokes malignant characteristics by activating the JAK/STAT pathway [38]. In NSCLC, KIF20A regulates the JNK pathway and acts as an oncogene, suggesting it has the potential to be a therapeutic target [39].

Eukaryotic cells are fundamentally dependent on vesicular tubular carriers, which transport membranes between organelles. Rab GTPases are a large family that recruits effector proteins to control membrane identity as well as motility, fusion, uncoating, and budding of vesicles and membrane identity. The spatiotemporal regulation of vesicle traffic is made possible by crosstalk between multiple Rab GTPases. Diseases like cancer, neurological disorders, and immunodeficiencies are linked to functional Rab pathway impairments [40]. The Rab family also regulates other cellular functions such as cytoskeletal transport and autophagy. Rab26 is associated with a cluster of synaptic vesicles in neurites and Atg16L1 may be a direct effector of Rab26 that can bind to Rab26 in GTP-bound form. Rab26 directs synaptic vesicles as well as secretory vesicles selectively into pre-autophagosomal structures [41]. Rab26 also regulates the permeability of endothelial cells; in air-liquid interface culture, it promotes the integrity of the adherent junction in an autophagy or macroautophagy-dependent manner. Rab26 interacts with Atg16L1 and maintains adherent junction stabilization through CDH5 dephosphorylation. Rab26-SRC signaling prevents vascular leakage [42]. Overexpressed Rab26 coalesces lysosomes to the perinuclear region, and the clustering of lysosomes initiates a redistribution of the mitochondria to subcellular neighborhoods. During differentiation, Rab26 coalesces the reorganization of the subcellular compartments by increasing the transcription of key effectors [43].

In this study, when cells were treated with miR-153-3p alone or in combination with doxorubicin, the intracellular vesicles decreased while doxorubicin alone had no such effect. Western blotting showed that Rab26 expression also decreased. Taken together, we demonstrated that miR-153-3p treatment enhanced the chemosensitivity of breast cancer cells to doxorubicin by downregulating KIF20A, leading to disrupted intracellular vesicle formation and doxorubicin-triggered cancer cell death by activating various pathways.

## 5. Conclusions

The findings of this study demonstrate that miR-153-3p upregulation is a crucial moderator that increases breast cancer cells’ Dox sensitivity and can directly target and downregulate KIF20A, which is essential for controlling the malignant process. Combination of miR-153-3p and Dox showed stronger effects of inhibiting breast cancer cells’ proliferation, migration and invasion and promoting apoptosis and S phase arrest than miR-153-3p and Dox alone. Consequently, miR-153-3p and Dox-related therapies may enable conventional chemotherapy and molecularly targeted therapy, providing a potential for targeting the formation of intracellular vesicles and addressing drug resistance issues currently present.

## Data Availability

The datasets used and/or analyzed during the current study are available from the corresponding author upon reasonable request.

## Supplementary Materials

The following supporting information can be downloaded at: https://www.mdpi.com/article/10.3390/cancers15061724/s1, Flie S1: Original blot.
