# Supplementary material for: Mir-153-3p Modulates the Breast Cancer Cells’ Chemosensitivity to Doxorubicin by Targeting KIF20A
Source: Cancers (Basel). 2023 Mar 11;15(6):1724. doi: 10.3390/cancers15061724 (PMC10046630; doi:10.3390/cancers15061724)
Supplement: Supplementary file 1 [file cancers-15-01724-s001.zip › cancers-2229450-supplementary.pdf]

Mir-153-3p Modulates the Breast Cancer Cells' Chemosensitivity to Doxorubicin by Targeting KIF20A

Western blots details

Figure 3: MCF-7 Cell line.

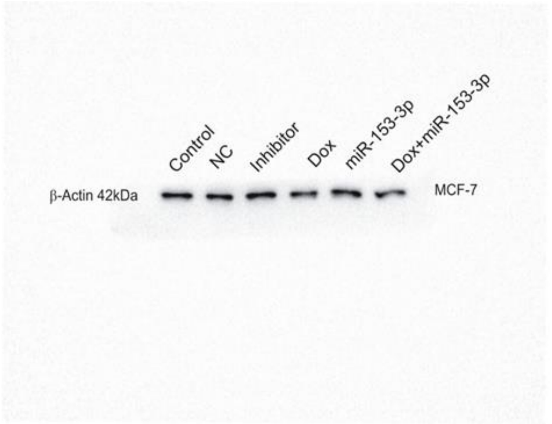

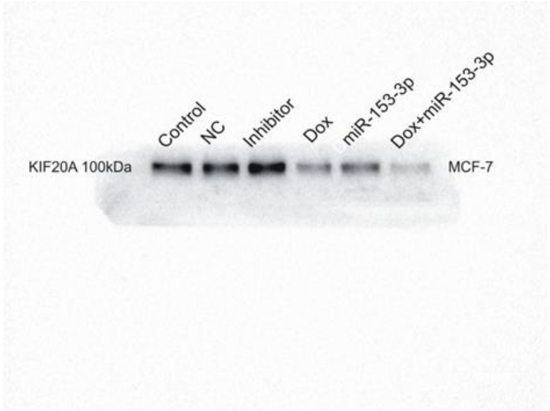

**KIF20A (MCF-7)**	
KIF20A/β-Actin Ratio in percentage	
Control	100
NC	98.89
Inhibitor	122.37
Dox	52.94
miR-153-3p	59.54
Dox+miR-153-3p	37.68

Figure 3: MDA-MB-231 Cell line.

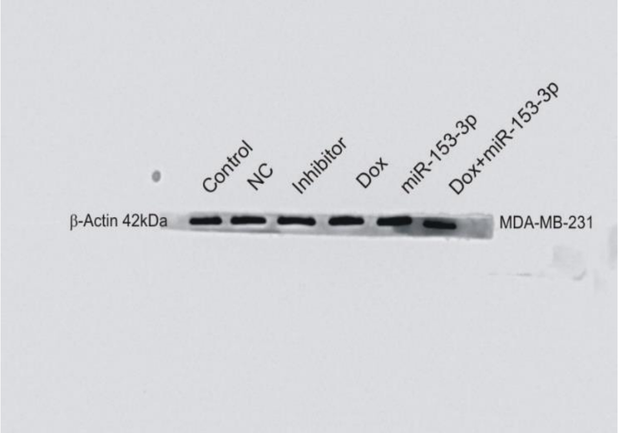

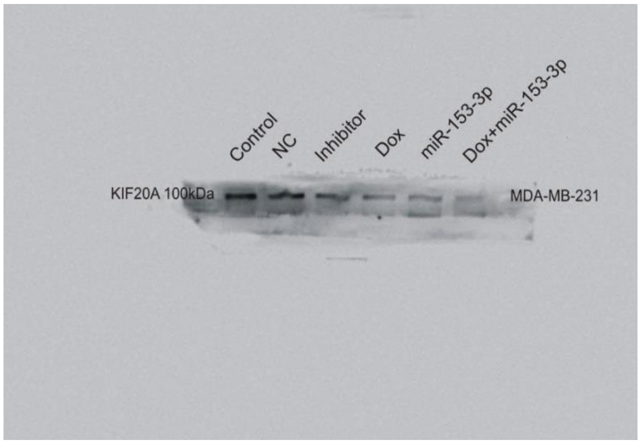

**KIF20A (MDA-MB-231)**	
KIF20A/β-Actin Ratio in percentage	
Control	100
NC	86.5695
Inhibitor	82.60747
Dox	54.0817
miR-153-3p	42.9246
Dox+miR-153-3p	36.4982

Figure 4:

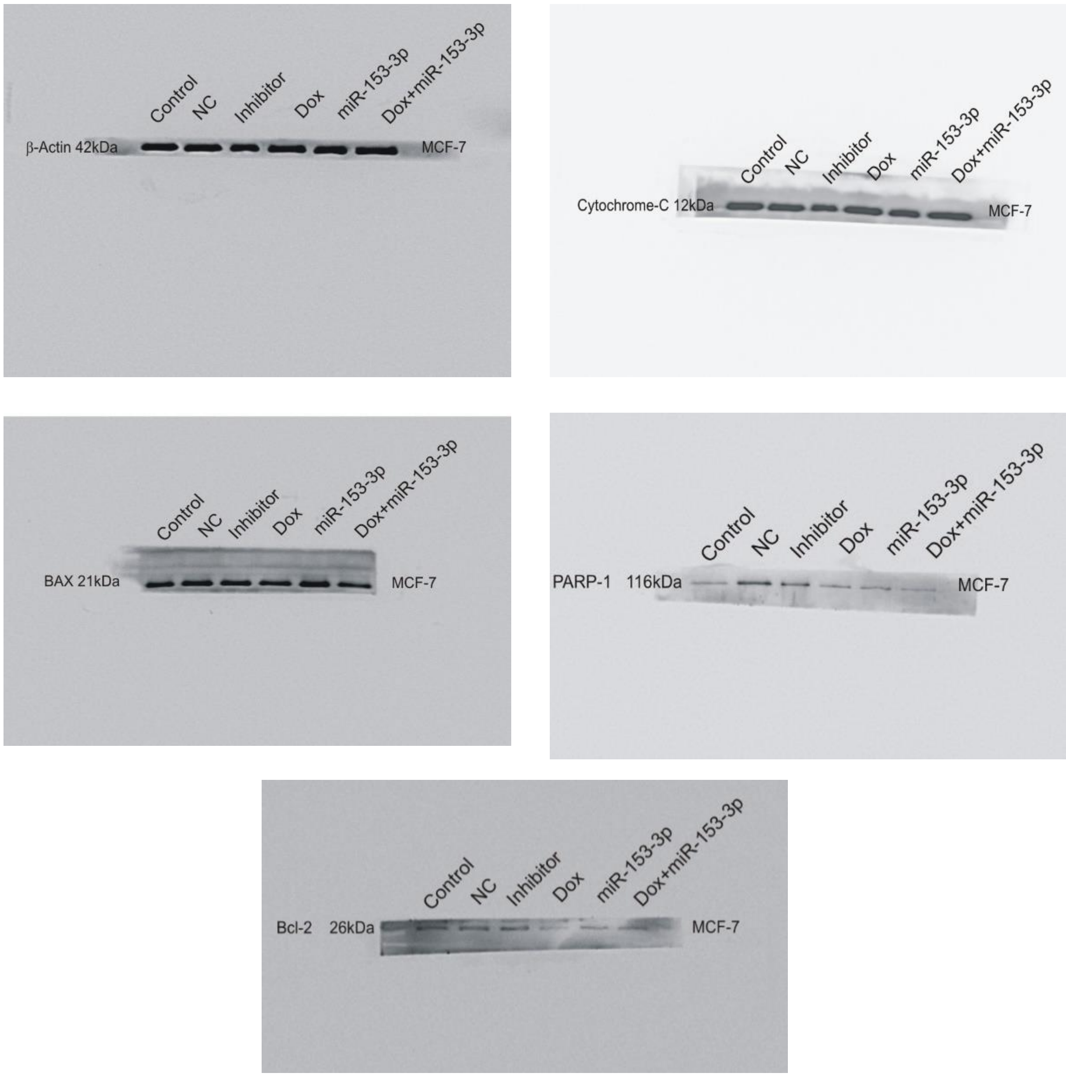

Control
NC
Inhibitor
Dox
miR-153-3p
Dox+miR-153-3p
β-Actin 42kDa
MCF-7
Control
NC
Inhibitor
Dox
miR-153-3p
Dox+miR-153-3p
Cytochrome-C 12kDa
MCF-7
Control
NC
Inhibitor
Dox
miR-153-3p
Dox+miR-153-3p
BAX 21kDa
MCF-7
Control
NC
Inhibitor
Dox
miR-153-3p
Dox+miR-153-3p
PARP-1 116kDa
MCF-7
Control
NC
Inhibitor
Dox
miR-153-3p
Dox+miR-153-3p
Bcl-2 26kDa
MCF-7

Cytochrome-C (MCF-7)	
Cytochrome-C /β-Actin Ratio in percentage	
Control	100
NC	106.91
Inhibitor	104.709
Dox	113.27
miR-153-3p	120.82
Dox+miR-153-3p	127.66

BAX (MCF-7)	
BAX /β-Actin Ratio in percentage	
Control	100
NC	91.2528
Inhibitor	91.8334
Dox	92.6008
miR-153-3p	105.5758333
Dox+miR-153-3p	97.6096

PARP1 (MCF-7)	
PARP1/β-Actin Ratio in percentage	
Control	100
NC	109.03
Inhibitor	109.12
Dox	94.51
miR-153-3p	64.88
Dox+miR-153-3p	49.90

Bcl-2 (MCF-7)	
Bcl-2/β-Actin Ratio in percentage	
Control	100
NC	78.85
Inhibitor	71.44
Dox	22.53
miR-153-3p	15.57
Dox+miR-153-3p	4.51

Figure 5:

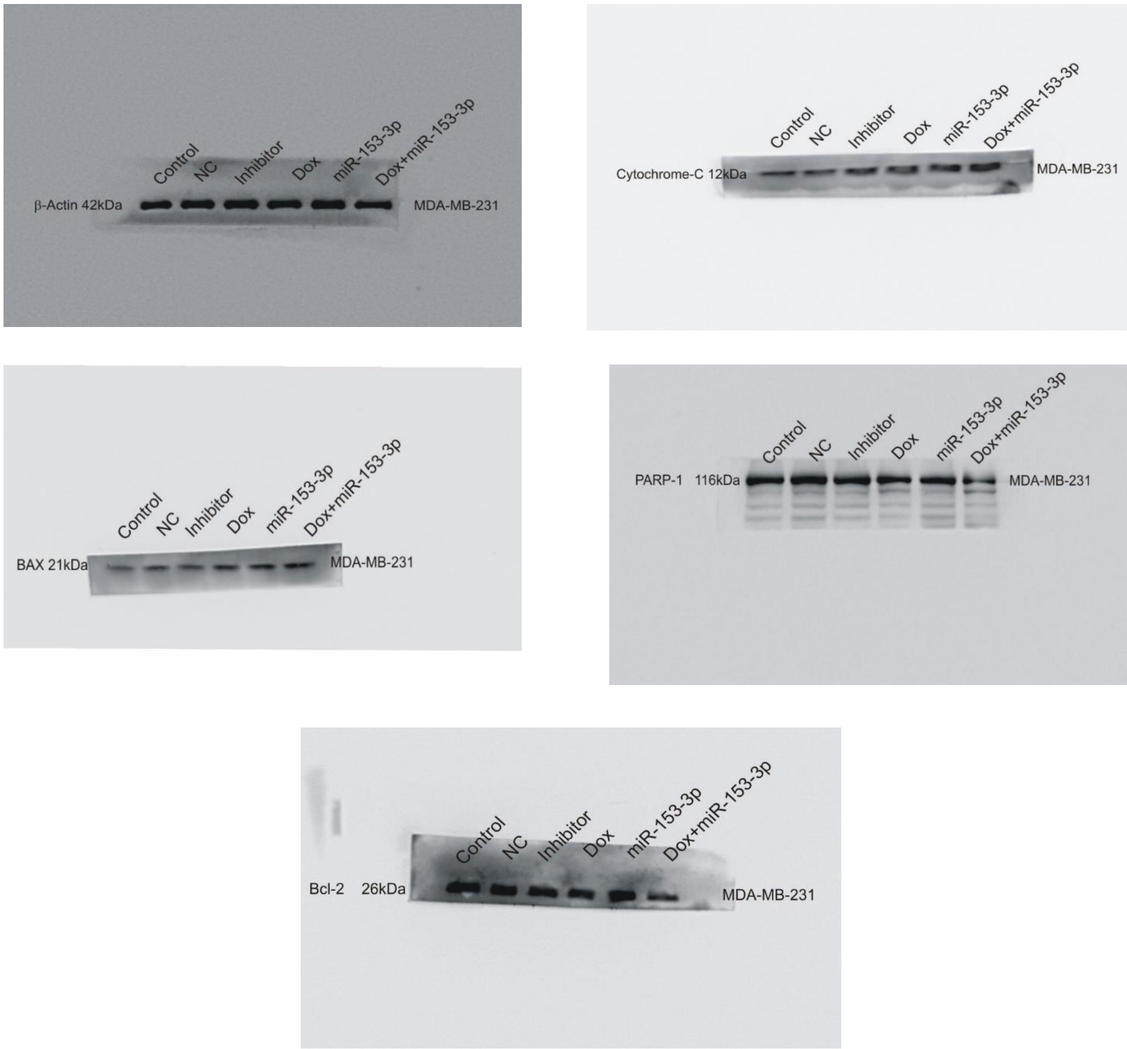

Cytochrome-C (MDA-MB-231)	
Cytochrome-C /β-Actin Ratio in percentage	
Control	100
NC	81.41
Inhibitor	84.7
Dox	97.71
miR-153-3p	111.5
Dox+miR-153-3p	122.5

BAX (MDA-MB-231)	
BAX /β-Actin Ratio in percentage	
Control	100
NC	138.5
Inhibitor	119.9
Dox	169.1
miR-153-3p	174.8
Dox+miR-153-3p	180.1

PARP1 (MDA-MB-231)	
PARP1/β-Actin Ratio in percentage	
Control	100
NC	82.12
Inhibitor	87.5
Dox	52.5
miR-153-3p	46.7
Dox+miR-153-3p	35.9

Bcl-2 (MDA-MB-231)	
Bcl-2/β-Actin Ratio in percentage	
Control	100
NC	86.9
Inhibitor	81.9
Dox	57.2
miR-153-3p	75.9
Dox+miR-153-3p	42.5

Figure 8:

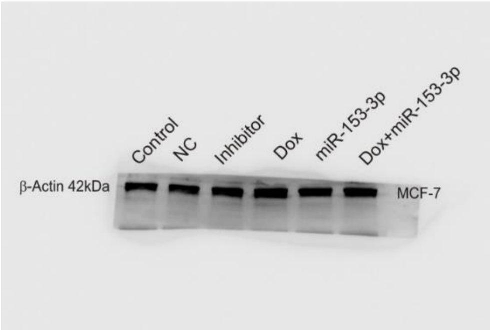

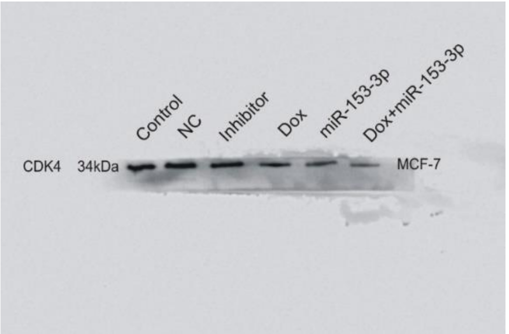

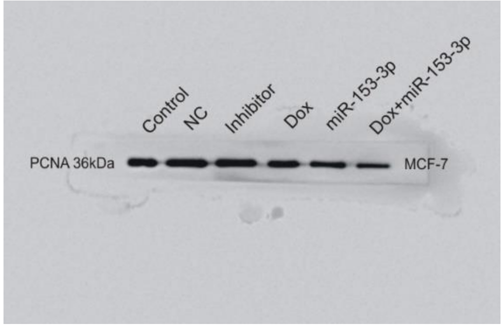

CDK4 (MCF-7)	
CDK4/β-Actin Ratio in percentage	
Control	100
NC	78.98945
Inhibitor	86.57644
Dox	58.49403
miR-153-3p	77.99591
Dox+miR-153-3p	53.93176

PCNA (MCF-7)	
PCNA /β-Actin Ratio in percentage	
Control	100
NC	119.2648
Inhibitor	115.6589
Dox	81.85907
miR-153-3p	70.72523
Dox+miR-153-3p	64.5823

Figure 9:

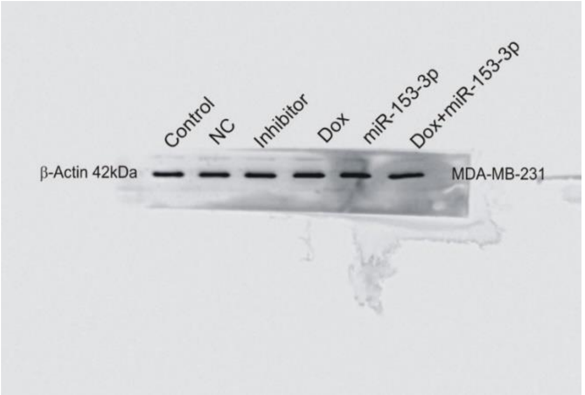

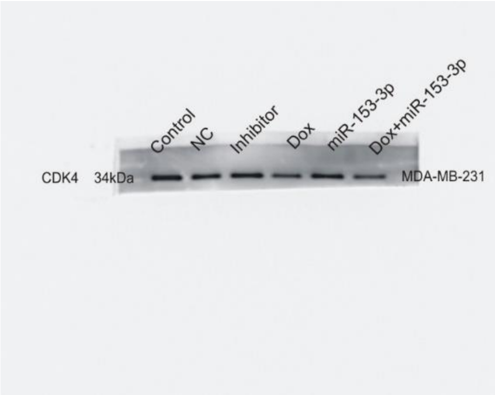

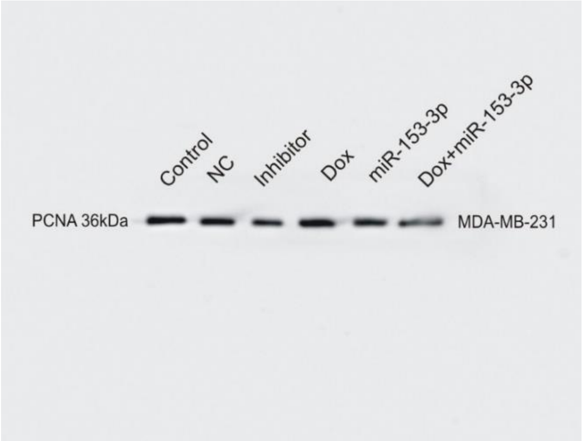

CDK4 (MDA-MB-231)	
CDK4/β-Actin Ratio in percentage	
Control	100
NC	109.40
Inhibitor	110.26
Dox	91.7
miR-153-3p	74.38
Dox+miR-153-3p	58.06

PCNA (MDA-MB-231)	
PCNA /β-Actin Ratio in percentage	
Control	100
NC	95.8
Inhibitor	93.5
Dox	95.6
miR-153-3p	74.4
Dox+miR-153-3p	65.5

Figure 10: MCF-7 Cell line

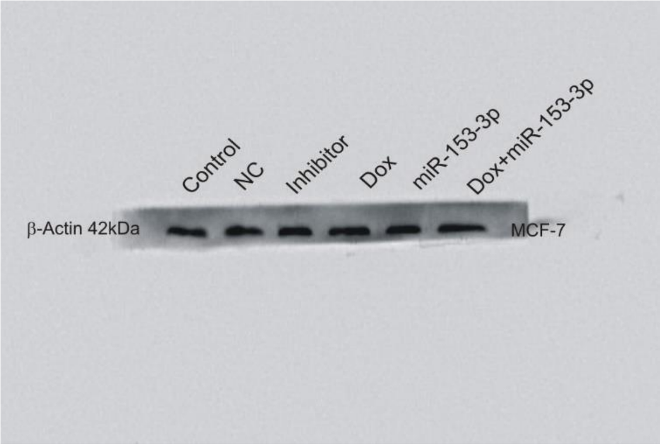

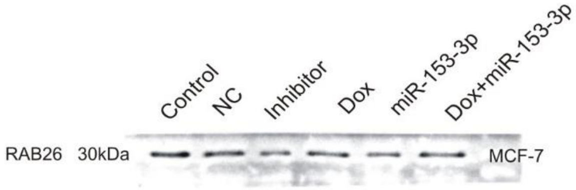

RAB26 (MCF-7)	
RAB26/β-Actin Ratio in percentage	
Control	100
NC	122.1881
Inhibitor	103.5713
Dox	84.7789
miR-153-3p	85.5201
Dox+miR-153-3p	75.9411

Figure 10: MDA-MB-231 Cell line

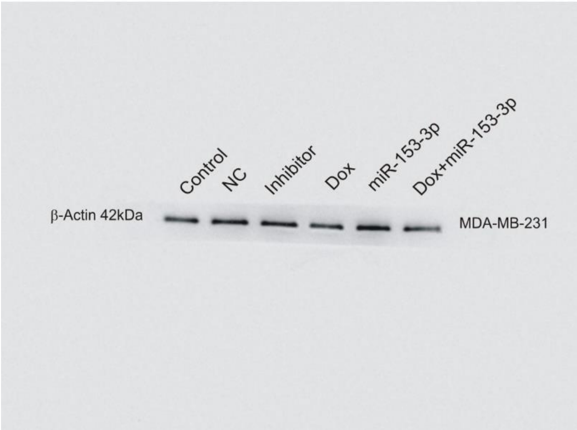

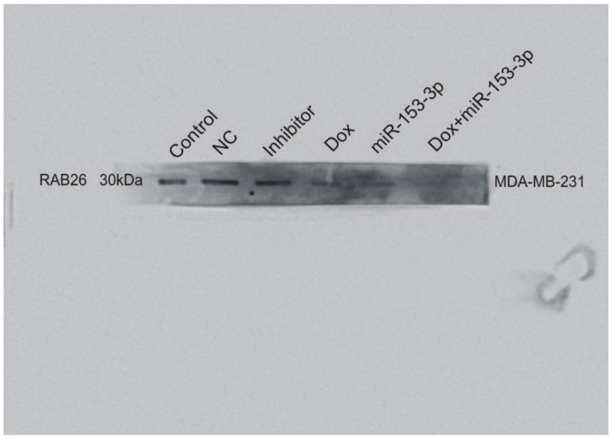

RAB26 (MDA-MB-231)	
RAB26/β-Actin Ratio in percentage	
Control	100
NC	115.5818
Inhibitor	99.6
Dox	47.8
miR-153-3p	77.2
Dox+miR-153-3p	37.3
